# Alkaloids with Activity against the Zika Virus Vector *Aedes aegypti* (L.)—Crinsarnine and Sarniensinol, Two New Crinine and Mesembrine Type Alkaloids Isolated from the South African Plant *Nerine sarniensis*

**DOI:** 10.3390/molecules21111432

**Published:** 2016-10-27

**Authors:** Marco Masi, Antonio Cala, Nurhayat Tabanca, Alessio Cimmino, Ivan R. Green, Jeffrey R. Bloomquist, Willem A. L. van Otterlo, Francisco A. Macias, Antonio Evidente

**Affiliations:** 1 Department of Chemical Sciences, University of Naples “Federico II”, Complesso Universitario Monte S. Angelo, Via Cintia 4, 80126 Napoli, Italy; marco.masi@unina.it (M.M.); antonio.cala@uca.es (A.C.); alessio.cimmino@unina.it (A.C.); 2 Allelopathy Group, Department of Organic Chemistry, School of Science, Institute of Biomolecules (INBIO), University of Cádiz, C/República Saharaui 7, 11510 Puerto Real, Cádiz, Spain; famacias@uca.es; 3 Department of Entomology and Nematology, Emerging Pathogens Institute, University of Florida, Gainesville, FL 32610, USA; ntabanca@ufl.edu@ufl.edu (N.T.); jbquist@epi.ufl.edu (J.R.B.); 4 U.S. Department of Agriculture-Agricultural Research Service, Subtropical Horticulture Research Station, 13601 Old Cutler Rd., Miami, FL 33158, USA; wvo@sun.ca.za; 5 Department of Chemistry and Polymer Science, University of Stellenbosch, Private Bag X1, Matieland, 7602 Stellenbosch, South Africa; irg@sun.ca.za

**Keywords:** *Nerine sarniensis*, Amaryllidaceae, mesembrine, crinine, crinsarnine, sarniensinol, bowdensine, Zika virus, *Aedes aegypti*

## Abstract

Two new Amaryllidaceae alkaloids, belonging to the mesembrine- and crinine-types, named crinsarnine (**1**) and sarniensinol (**2**), were isolated from the dried bulbs of *Nerine sarniensis* together with bowdensine (**3**), sarniensine (**4**), hippadine (**5**) and 1-*O*-acetyl-lycorine (**6**). Crinsarnine (**1**) and sarniensinol (**2**) were characterized using spectroscopic and chiroptical methods as (1*S*,2*S*,4a*R*,10b*S*)-2,7-dimethoxy-1,2,3,4,4a,6-hexahydro-5,11b-ethano[1,3]dioxolo-[4,5-*j*]phenanthridin-1-yl acetate and (6-(3a*R*,4*Z*,6*S*,7a*S*)-6-methoxy-1-methyl-2,3,3a,6,7,7a-hexa-hydro-1*H*-indol-3a-yl)benzo[*d*][1,3]dioxol-5-yl)methanol, respectively. Furthermore, the complete spectroscopic characterization of bowdensine (**3**) is reported for the first time. Compounds **1**–**6** were evaluated against the Orlando reference strain of *Aedes aegypti*. None of compounds showed mortality against 1st instar *Ae. aegypti* larvae at the concentrations tested. In adult topical bioassays, only **1** displayed adulticidal activity with an LD_50_ = 2.29 ± 0.049 μg/mosquito. As regards the structure-activity relationship, the pretazettine and crinine scaffold in **2** and **4** and in **1** and **3** respectively, proved to be important for their activity, while the pyrrole[*de*]phenanthridine scaffold present in **5** and **6** was important for their reactivity. Among the pretazettine group compounds, opening of the B ring or the presence of a B ring lactone as well as the *trans*-stereochemistry of the A/B ring junction, appears to be important for activity, while in crinine-type alkaloids, the substituent at C-2 seems to play a role in their activity.

## 1. Introduction

*Aedes aegypti* (L.), the yellow fever mosquito, is the primary vector of dengue and yellow fever viruses. The current Zika virus outbreak is proving to be most concerning since it could easily become a potential threat to international public health safety if not addressed very soon. The virus causes microcephaly and other serious brain anomalies during pregnancy [1]. Mosquito control is one of the main methods used to reduce the spread of the virus. Personal protection, insecticidal treatment regimens against larval and adult mosquitoes and reduction or complete destruction of mosquito habitats are among the main methods to reduce such vector-borne diseases [2]. Biological control strategies such as use of the maternally inherited bacterium *Wolbachia pipientis* to reduce transmission of viruses in *Ae. aegypti* mosquitoes [3] and larvivorous fish represents an alternative to controlling the *Ae. aegypti* larvae and pupae [4]. Moreover, extensive use of chemical insecticides for mosquito control has resulted in the development of pesticide resistance and unwanted environmental effects [5]. Hence, plant based secondary metabolites have attracted considerable interest in pest management programs as insecticides and repellents. Among them, the Amaryllidaceae plants have demonstrated to be a good reservoir of alkaloids with a large spectrum of biological activity [6,7]. In fact, in the last century, Amaryllidaceae plants have stimulated renewed interest in the scientific community due to the high number of bioactive metabolites isolated from them. There are over 1000 species of these plants belonging to 60 genera spread widely around the World. In South Africa, these bulbous plants are grown for their ornamental value and have been used in the past in folk medicine [8,9].

One of the most remarkable groups of natural products isolated from these plants are alkaloids which present a wide variety of most interesting biological activities. They have been classified into twelve groups on the basis of their different carbon skeletons [6,7]. To date, literally hundreds of new structurally diverse alkaloids [10] have been found and many of these have shown promising anticancer activities, viz., lycorine, isolated for the first time in 1877 is one of the most widely studied alkaloids for its biological activity [6]. Numerous other Amaryllidaceae alkaloids from different groups, which include crinamine and the recently discovered pretazettine-type, jonquailine, exhibit interesting anticancer activities [11]. Among the general Amaryllidaceae alkaloids, galanthamine has proved most promising and is currently being used as a drug for the treatment of Alzheimer’s disease [12].

**Figure 1:**
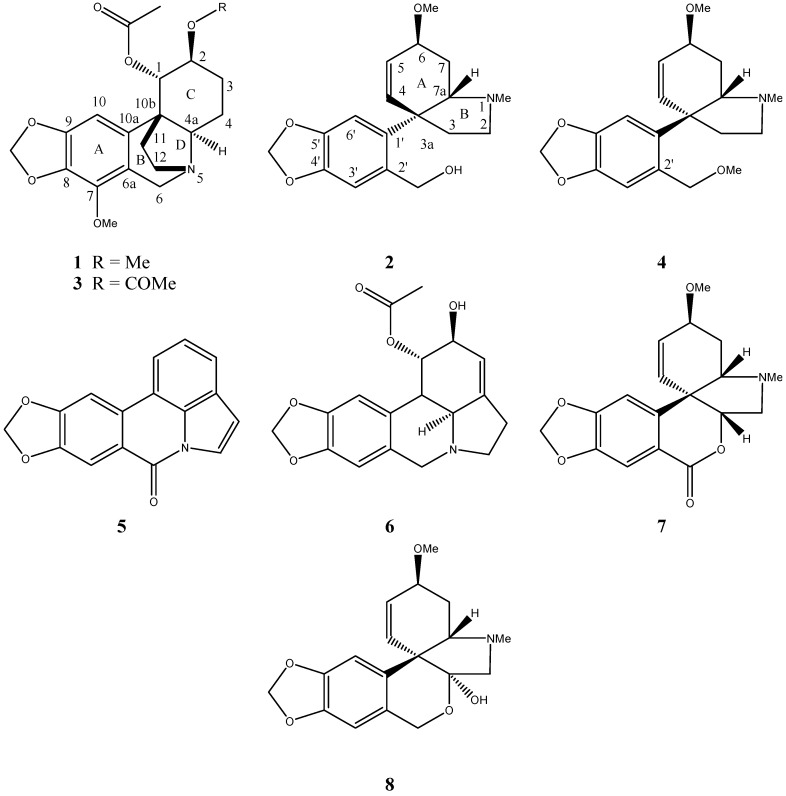
Structures of crinsarnine (**1**), sarniensinol (**2**), bowdensine (**3**), sarniensine (**4**), hippadine (**5**), 1-*O*-acetyl-lycorine (**6**), 3-epimacronine (**7**) and tazettine (**8**).

*Nerine sarniensis* cv. Red is a species restricted to the Western Cape of South Africa [13] and isolation of its alkaloids has been largely unexplored and thus very little is known about these plant metabolites. The first study of this plant was reported by Boit [14], and was followed by a second study by our research group [15], who described the isolation of a new mesembrine-type alkaloid, sarniensine (**4**, Figure 1), along with tazettine (**8**, Figure 1), the main alkaloid, lycorine, and 3-epimacronine (**7**, Figure 1) from *N. sarniensis* bulbs. Sarniensine (**4**) showed adulticidal activity against *Aedes aegypti*, a mosquito which is the major vector for dengue, yellow fever and the presently alarming Zika virus [15].

The current paper reports on the isolation and structural determination of additional alkaloids isolated from *N. sarniensis*. Two new alkaloids, named crinsarnine (**1**) and sarniensinol (**2**), were isolated together with bowdensine (**3**), sarniensine (**4**), hippadine (**5**) and 1-*O*-acetyl-lycorine (**6**). The latter isolated compounds are now reported for the first time from *N. sarniensis*, along with the insecticidal activity of alkaloids **1**–**8** against *Ae. aegypti* mosquitoes. Since rather sparse information was found about the spectral characterization of **3** [16,17], the complete NMR spectroscopic data for this alkaloid is also reported here for completeness.

## 2. Results and Discussion

The known alkaloids bowdensine (**3**), hippadine (**5**) and 1-*O*-acetyl-lycorine (**6**) were isolated for the first time from *N. sarniensis*, and were identified by comparison of their spectroscopic properties with those previously reported in the literature for **3** [16,17], **5** [18] and **6** [19].

The NMR spectra showed that crinsarnine (**1**) and sarniensinol (**2**) belong to two different subgroups of Amaryllidaceae alkaloids (Figure 1). Crinsarnine (**1**) had a molecular formula of C_20_H_25_NO_6_ as deduced from its HR ESIMS spectrum, consistent with nine hydrogen deficiencies. The ^1^H- and ^13^C-NMR spectra (Table 1) showed a signal system typical of crinine-type alkaloids. In fact, the ^1^H-NMR spectrum showed signals of the methylenedioxy system, the single aromatic proton of a pentasubstituted A ring, the AB system of the nitrogen methylene linked to ring A at δ 5.84 (d, *J* = 1.3 Hz) and 5.83 (d, *J* = 1.3 Hz)/100.5 (O-CH_2_-O), 6.24 (s)/97.5 (HC-10) and 4.19 (d, *J* = 17.3 Hz) and 3.77 (d, *J* = 17.3 Hz)/58.3 (H_2_C-6). In addition, the same spectrum illustrated complex spin systems due to the substituted C ring and an ethane-bridge in the D ring. In fact, suitably 1,2,5-substituted W and A_2_B_2_ systems [20,21] were observed for the protons of the C ring and the ethane-group respectively, in which the latter connected the B/C bridgehead carbon C-10b to the tertiary nitrogen atom of the D ring. The doublet (*J* = 3.7 Hz), observed at δ 5.14, was assigned to the esterified methine (HC-1), and was coupled in the COSY spectrum [22] with the proton of the adjacent oxygenated methine (HC-2), which appeared as a broad single peak at δ 3.96. H-2 was coupled with the broad doublet (*J* = 13.6 Hz) and broad triplet (*J* = 13.6 Hz) at δ 2.09 and 1.33, respectively, which were assigned to the adjacent H_2_C-3 methylene group. These latter protons were coupled with those of the adjacent H_2_C-4 methylene group, appearing as a triple doublet (*J* = 13.6 and 4.8 Hz) and a multiplet at δ 1.66 and 1.57, respectively. These, in turn, were coupled with the double doublet (*J* = 13.6 and 4.8 Hz) of H-4a observed at δ 3.00 [21]. Considering that ring C assumes a hemi-chair like conformation, the coupling constants of these signals allowed the authors to locate the signals of H-3B and H-4B and H-4a as α-, β- and α-axial, as well as H-2, H-3A and H-4A as α-, β- and α-equatorial. Both ^1^H- and ^13^C-NMR spectra also showed the typical singlets of an acetoxy group, confirmed by the corresponding C=O bands observed in the IR spectrum [23] appearing at δ 170.9 (COOCH_3_) and 2.22/21.6 (COOCH_3_) and those of two methoxy groups at δ 3.96/59.1 (OCH_3_) and 3.31/57.7 (OCH_3_) [21,24]. The acetoxy group was located at C-1 while the two methoxy groups were located at C-2 and C-7 as deduced from the HMBC spectrum [22]. The relative stereochemistry at C-1 is assigned as β-equatorial and was deduced by the absence of any correlation observed in the NOESY spectrum [22] between both H-1 and H-2 as well as between the 1-acetoxy and 2-methoxy groups which thus located these two protons in the 1-α- and 2-β-axial orientations, respectively. The couplings observed in the HSQC spectrum [22] facilitated the assignments of the chemical shifts to all protonated carbons. Thus, the signals at δ 76.8, 75.8, 68.2, 52.3, 37.3, 24.4 and 20.4 were assigned to C-1, C-2, C-4a, C-12, C-11, C-3 and C-4. The other couplings observed in the HMBC spectrum allowed assignment of the remaining aromatic quaternary carbons. In particular, the coupling between C-6a and H_2_-6 and H-10, C-7 and H_2_-6, H-10 and O-CH_2_-O, C-8 and H_2_-6, C-9 and O-CH_2_-O, H-10 and OMe (C-7), C-10a and H_2_-6, H-10 and O-CH_2_-O, allowed the assignment of the signals at δ 148.1, 141.3, 140.3, 133.4 and 116.7 to C-7, C-8, C-9, C-10a and C-6a. [24]. Thus, chemical shifts were assigned to all the protons and corresponding carbons as reported in Table 1 and **1** was formulated as (1*S*,2*S*,4a*R*)-2,7-dimethoxy-1,2,3,4,4a,6-hexahydro-5,11b-ethano[1,3]dioxolo[4,5-*j*]phenanthridin-1-yl acetate. The structure assigned to **1** was supported by all the HMBC couplings reported in Table 1 and from the data of its HR ESIMS spectrum, which showed the pseudomolecular ion [M + H]^+^ at *m*/*z* 376.1766.

**Figure 2:**
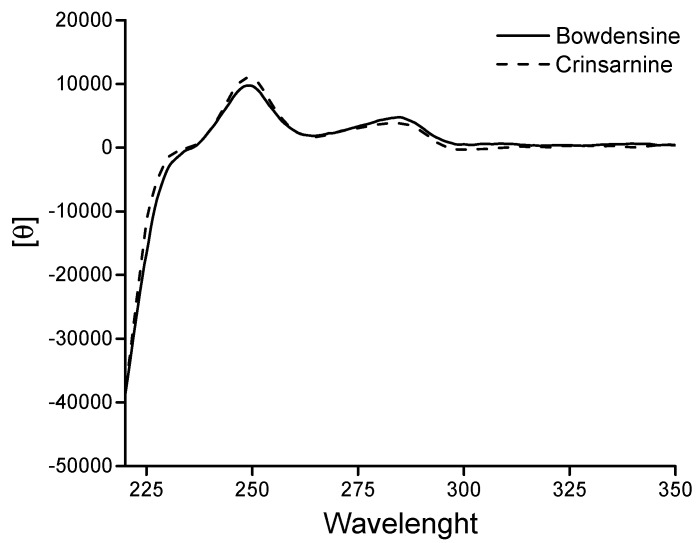
ECD spectra of crinsarnine (**1**) and bowdensine (**3**) recorded in MeOH.

The (1*S*,2*S*,4a*R*,10b*S*) absolute configurations of crinsarnine (**1**) and bowdensine (**3**) were assigned by comparing their ECD spectra with those reported in literature for the closely similar crinine alkaloids [25]. As expected, the ECD spectra of **1** and **3** recorded under the same conditions (Figure 2) practically overlapped each other, supporting the assigned absolute configurations assigned to them.

In the case of acetate **3,** only partial ^1^H-NMR spectral data was available for a comparison [14] and thus its ^1^H- and ^13^C-NMR spectral data (Table 1), also supported by the respective COSY, HSQC and HMBC spectra, are now fully reported here for completeness and to confirm the assigned structure.

Sarniensinol (**2**) is here reported as the second new alkaloid isolated from the crude organic extract of *N. sarniensis*. Comparison of its ^1^H- and ^13^C-NMR spectra (Table 2) with that of sarniensine (**4**, Figure 1, recorded under the same conditions), a pretazettine type alkaloid recently isolated from the same plant [15], illustrated an overlapping in all the spectral regions. One most obvious difference was the fact that **2** lacked the signal for the methoxy group of the methoxy side chain at C-2′ in **4**. This difference allowed for assigning the chemical shifts to all the protons and corresponding carbons of **2** on the basis of the clear couplings observed in the respective COSY, HSQC and HMBC spectra and are reported in Table 2. Thus, **2** was formulated as (6-(6-methoxy-1-methyl-2,3,3a,6,7,7a-hexahydro-1*H*-indol-3a-yl)benzo[*d*][1,3]dioxol-5-yl)methanol. The structure assigned to **2** was confirmed from the data of its HR ESIMS spectrum which showed the pseudomolecular ion [M + H]^+^ at *m*/*z* 318.1712.

**Figure 3:**
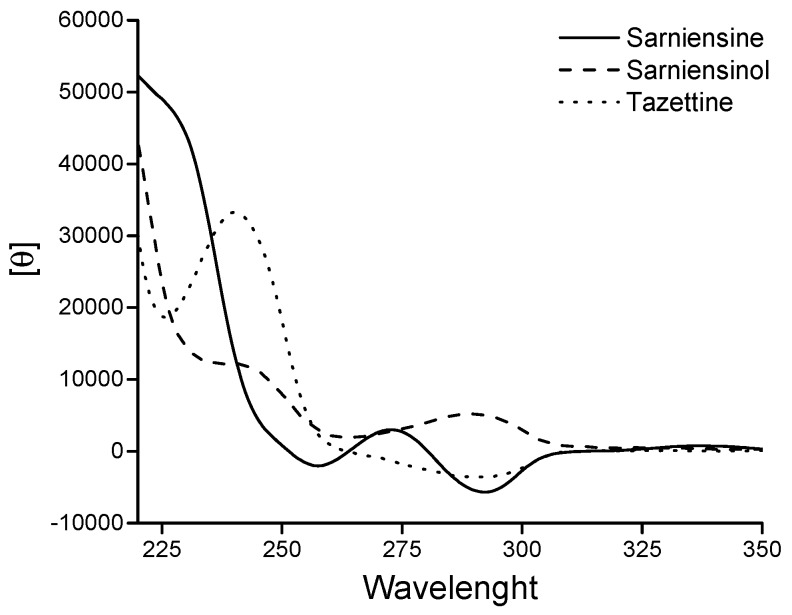
ECD spectra of sarniensinol (**2**), sarniensine (**4**) and tazettine recorded in MeOH.

The absolute configuration 3a*S*,4*Z*,6*S*,7a*S* was assigned to **2** by comparing its ECD spectrum with that of sarniensine (**4**) recorded under the same conditions (Figure 3) and previously reported [15] as well as with spectra reported in the literature for egonine and tazettadiol isolated from *Hippeastrum equestre* [26]. A notable discovery was that the alkaloids sarniensinol (**2**), sarniensine (**4**), egonine and tazettadiol all displayed similar profiles in the CD spectra in the region between 225 and 260 nm while sarniensinol (**2**) displayed an opposite Cotton effect with respect to the other three alkaloids, vide supra, at about 290 nm. This observation suggests that the A/B ring fusion for **2** is *trans* and opposite to that of egonine, tazettadiol and sarniensine (**4**) which are all *cis*. This opposite stereochemistry is probably the main reason for the significant different value for the coupling between the two olefinic protons H-4 and H-5 in ring A appearing as broad singlets compared to the same protons observed in sarniensine (**4**) as two doublets with *J* = 10.5 Hz [15] and in egonine as a doublets of double doublets with *J* = 10.6, 2.1 and 1.8 Hz [26].

In the current study, the isolated compounds crinsarnine (**1**), sarniensinol (**2**), bowdensine (**3**), hippadine (**5**) and 1-*O*-acetyl-lycorine (**6**) were evaluated for their mosquitocidal activity against *Ae. aegypti* and their activities were compared to those of sarniensine (**4**), 3-epimacronine (**7**) and tazettine (**8**), as previously reported [15]. Initial larval activity testing indicated that none of these compounds **1**–**3**, **5** and **6** showed enough mortality to be further studied for their dose response bioassays at the prescreened dosages of 1.00, 0.50, 0.25 and 0.10 μg·μL^−1^ (Table 3).

In adult topical bioassays on *Ae. aegypti*, compound **1** proved to be the most active, with 97% ± 6% mortality at the test dose of 5 μg·mosquito^−1^ in three replicated experiments, while the remaining compounds produced 23% (**6**) and 33% (**2**, **3**, **5**) mortality. Compound **1** was further evaluated and had an LD_50_ value of 2.3 μg/mosquito against adult female *Ae. aegypti* (Table 4).

The results (Table 4), also showed that the pretazettine and crinine carbon skeleton present in **2**, **4** and **7**, and in **1** and **3** respectively, are very important for the activity with respect to pyrrole[*de*]phenanthridine ring present in **5** and **6**. Among the pretazettine group, compound **4** was more active than either **7** or **2**, which suggests that opening of the B ring or the presence of a lactone B ring supports our previous findings [15] where we suggested that these same structural features played a major role in the biological activity of the isolates of *N. sarniensis*. Furthermore, in the same group of alkaloids, the stereochemistry of the A/B ring junction, being *trans* in **2** and *cis* in **4** and 7, also appear to play a role. Among the crinine type alkaloids, compound **1** was the most active metabolite and more so than **3**, which suggests that the substituent at C-2 certainly plays a significant role in the activity profile of the molecule.

## 3. Materials and Methods

### 3.1. General Information

ECD and absorption spectra were measured in MeOH at room temperature on a J-815 spectropolarimeter (JASCO, Tokyo, Japan), using 1 cm cells. Optical rotations were measured in CHCl_3_ on a JASCO P-1010 polarimeter; IR spectra were recorded as deposit glass film on a Thermo Electron Corporation Nicolet 5700 FT-IR (Thermo, Waltham, MA, USA) spectrometer and UV spectra were measured in MeOH on a JASCO V-530 spectrophotometer. ^1^H- and ^13^C-NMR spectra were recorded at 400/100 MHz in CDCl_3_ on Bruker (Karlsruhe, Germany) spectrometers. The residual peak of the solvent was used as internal standard in each case. Carbon multiplicities were determined by DEPT spectra [22]. DEPT, COSY-45, HSQC, HMBC, and NOESY experiments were performed using the Bruker software programs. HRESIMS and ESI spectra were recorded on Shimadzu (Kyoto, Japan) LCMS-IT-TOF Mass Spectrometer and Agilent (Milan, Italy) 6230 Accurate-Mass TOF LC/MS instruments. Analytical and preparative TLC were performed on silica gel (Merck, Darmstadt, Germany, Kieselgel 60, F254, 0.25 and 0.5 mm respectively) plates. The spots were visualized by exposure to UV radiation (253), or iodine vapour. Colum chromatography (CC) was performed using silica gel (Merck, Kieselgel 60, 0.063–0.200 mm).

### 3.2. Plant Material

Bulbs of *Nerine sarniensis* were purchased from the South African Bulb Company and three live specimens are growing in the Botanical Garden at the University of Stellenbosch, Stellenbosch, South Africa.

### 3.3. Extraction and Purification of N. sarniensis Bulbs Extract

The organic extract of *N. sarniensis* dried bulbs was obtained following the procedure previously described [15]. Briefly, dried and minced bulbs (158.7 g) were macerated with 1% H_2_SO_4_, (2 × 850 mL) overnight at 2 °C, filtered through cloth and basified to pH 8 with 12 N NaOH. The aqueous solution was extracted with EtOAc (6 × 600 mL), the organic extracts were combined, dried (anhydrous Na_2_SO_4_) and evaporated under vacuum to provide a brown oil (1.6 g). The extract was fractionated via a bioguided process by column chromatography using CHCl_3_–EtOAc–MeOH (2:2:1) as eluent, followed by CHCl_3_–EtOAc–MeOH (85:10:5), MeOH and finally with MeOH–H_2_O (95:5) affording twelve groups of homogeneous fractions (AF1-AF12). As reported previously, purification of the AF2 residue (195 mg) yielded sarniensine (**4**). The residue of fraction AF1 (113 mg) was refractionated by column chromatography and eluted with CHCl_3_–*i*-PrOH (95:5) and finally with MeOH to give nine fractions (BF1-BF9). The residue of BF2 (16.6 mg) was further purified by analytical TLC using *n*-hexane–EtOAc (7:3) as eluent to afford hippadine (**5**, 2.1 mg, *R*_f_ = 0.57) as an amorphous solid. The residue of BF5 (11.3 mg) was purified by analytical TLC using CHCl_3_–*i*-PrOH (95:5) as eluent, to yield 3-epimacronine (**7**, 2.6 mg, *R*_f_ = 0.58) as an amorphous solid. Fraction AF3 residue (164 mg) was purified by column chromatography, using CHCl_3_–MeOH (9:1) as eluent yielding seven fractions viz., (CF1-CF7). Fractions CF5 and CF6 contained mainly lycorine. The residue of CF2 (1.5 mg) was identified as sarniensine (**4**, *R*_f_ = 0.88, column eluent). The residue of CF3 (79 mg) was further purified by preparative TLC and eluted with EtOAc–MeOH–H_2_O (90:7:3) as eluent, followed by a second purification with CHCl_3_–*i*-PrOH (9:1) as eluent to afford 1-*O*-acetyl-lycorine (**6**, 4 mg), as white crystals, bowdensine (**3**, 12 mg) and crinsarnine (**1**, 4.9 mg) as amorphous solids (*R*_f_ = 0.41, 0.32 and 0.27 respectfully). The residue of CF4 (17.1 mg) was further purified by TLC with EtOAc–MeOH–H_2_O (85:10:5) as eluent, to afford tazettine (**8**, 1.2 mg) and sarniensinol (**2**, 2.2 mg) as amorphous solids.

### 3.4. Characterization of Compounds

*Crinsarnine* (**1**): UV (log ε) λ_max_ 285 (2.4), 235 (3.5), 210 (4.3); CD ([θ]_λ_): see Figure 2; ${\left[\alpha \right]}_{D}^{25}$: +1.8° (*c* = 0.49); IR ν_max_ 2926.1, 2853.0, 1734.7, 1616.1, 1477.2, 1234.1, 1091.5, 1044.5 cm^−1^; ^1^H- and ^13^C-NMR data; see Table 1; HRESIMS *m*/*z*: 376.1766 [M + H]^+^ (calcd. for C_20_H_26_NO_6_, 376.1760).

*Sarniensinol* (**2**): UV (log ε) λ_max_ 289 (3.0), 242 (3.3), 207 (4.2); CD ([θ]_λ_): see Figure 3; ${\left[\alpha \right]}_{D}^{25}$: +50.0° (*c* = 0.22); IR ν_max_ 3660.8, 2922.9, 1655.5, 1512.5, 1252.5, 1097.2 cm^−1^; ^1^H- and ^13^C-NMR data; see Table 2; HRESIMS *m*/*z*: 318.1712 [M + H]^+^ (calcd. for C_18_H_24_NO_4_, 318.1705).

*Bowdensine* (**3**): CD ([θ]_λ_): see Figure 2; ${\left[\alpha \right]}_{D}^{25}$: +5.3° (*c* = 0.46); ^1^H- and ^13^C-NMR data; see Table 2; HRESIMS *m*/*z*: 404.1713 [M + H]^+^ (calcd for C_21_H_26_NO_7_, 404.1709).

### 3.5. Mosquitoes and Bioassay

*Aedes aegypti* larvae and adults (Orlando, FL, USA, 1952) used in this study were sourced from laboratory colonies maintained at the USDA-ARS (CMAVE, Gainesville, FL, USA). Rearing larvae from eggs has been described previously [15,27,28]. Larvicidal activity was performed as described previously [15,27]. Embryonated eggs were hatched overnight from oviposition papers in deionized water with a larval diet. Test compounds were initially diluted in DMSO to make 100 µg/µL. Mortality was determined in the larval assays at four different concentrations (1.0, 0.5, 0.25, and 0.1 µg/µL) in a final volume of 200 µL of larval rearing media that contained five 1st. For each assay, a positive control of permethrin stock and a negative control of DMSO was included. Assays were repeated at least three times on separate days.

To determine the toxicity of each sample against female *Ae. aegypti*, samples were initially diluted to a 10% DMSO solution that was subsequently serially diluted 1:10 in acetone. Mosquitoes were cold anesthetized on ice and groups of 10 females sorted into individual plastic cups. Application of 0.5 µL of the appropriate dilution of the test alkaloid was made by repeater pipettor (PB600, Hamilton, Reno, NV, USA) with a 25 µL blunt tip glass syringe (Hamilton 7100 series). Mortality was scored 24 h after application. Tests were replicated three times. Compounds showing mortality >80% were further assessed for LD_50_ dose-response bioassays, as described previously [15]. Permethrin was used as positive control and acetone alone as negative control in all bioassays. Mortality data was analyzed in SigmaPlot.v13 using the best fit sigmoidal plot with the minimum and maximum constrained to 0% and 100%, respectively.

## 4. Conclusions

Two new Amaryllidaceae alkaloids, belonging to the mesembrine and crinine type, named crinsarnine (**1**) and sarniensinol (**2**) were isolated from the dried bulbs of *N. sarniensis* together with bowdensine (**3**), sarniensine (**4**), hippadine (**5**) and 1-*O*-acetyl-lycorine (**6**). Crinsarnine (**1**) was the only one that showed adulticidal activity and appears to possess potential as a natural based insecticide against *Ae. aegypti*, the major vector for dengue, yellow fever and the Zika virus. A more integrated and environmentally friendly insect control system requires models for the development of new insecticides based on natural strategies. The results described here illustrate that natural products, and particularly the Amaryllidaceae alkaloids, are valuable resources which need to be investigated further as natural pesticides against *Ae. aegypti*, the major vector for dengue, yellow fever and of course the Zika virus.

## Figures and Tables

**Table 1:** ^1^H-, ^13^C-NMR and HMBC spectroscopic data of crinsarnine (**1**) and bowdensine (**3**) ^a,b^.

No.	1			3		
	^13^C ^c^	^1^H (*J* in Hz)	HMBC	^13^C ^c^	^1^H (*J* in Hz)	HMBC
1	76.8	5.14 (1H) d (3.7)	H-11	74.1	5.32 (1H) d (4.4)	H-2, H-3A, H-11A, COOCH_3_
2	75.8	3.96 (1H) br s ^d^	OMe (2)	68.3	5.57 (1H) br s	H-1, H-3B, COOCH_3_
3	24.4	2.09 (1H) br d (13.6) 1.33 (1H) br t (13.6)	H-4B, H-4a	26.4	1.94 (1H) br d (14.4) 1.57 (1H) br dd (14.4, 11.4)	H-4B
4	20.4	1.66 (1H) td (13.6, 4.8) 1.57 (1H) m	H-3B, H-4a	21.2	2.06 (1H) td (11.4, 5.5) 1.72 (1H) m	H_2_-3, H-4a
4a	68.4	3.00 (1H) dd (13.6, 4.8)	H-3B, H-4B, H-6B, H-11B, H-12A	68.2	3.03 (1H) dd (5.5, 11.4)	H-3B, H_2_-4, H-6B, H-11B, H-12A
6	58.3	4.19 (1H) d (17.3) 3.77 (1H) d (17.3)	4a, H_2_-12	58.4	4.18 (1H) d (17.5) 3.77 (1H) d (17.5)	H-4a, H-10, H-12A, H-12B
6a	116.7		H_2_-6, H-10	117.0		H-6A, H-6B, H-10
7	148.1		H_2_-6, H-10, OCH_2_O	148.1		H-10
8	141.3		H-1, H-4a, H_2_-6, H_2_-11	141.0		H_2_-6
9	140.3		H_2_-6, H-10, OMe (7)	140.3		H-10, OMe
10	97.5	6.24 (1H) s		97.4	6.18 (1H) s	
10a	133.4		H_2_-6, H-10, OCH_2_O	133.5		H_2_-6, H-10, OCH_2_O
10b	124.5			129.7		
11	37.3	2.03 (1H) ddd (16.0, 9.2, 4.3) 2.92 (1H) ddd (16.0, 11.6, 6.3)	H-1, H-4a,H-6A, H-12	37.3	2.74 (1H) ddd (13.0, 11.0, 5.5) 2.03 (1H) ddd (13.0, 9.0, 6.0)	H-1, H-4a, H-6A, H-12A
12	52.3	2.79 (1H) ddd (14.6, 11.6, 4.3) 3.39 (1H) ddd (14.6, 9.2, 6.3)	H-4a, H_2_-6, H-11A	52.3	3.42 (1H) ddd (12.4, 9.0, 5.5) 2.82 (1H) ddd (12.4, 11.0, 6.0)	H-4a, H_2_-6, H-11A
OCH_2_O	100.5	5.83 (1H) d (1.3) 5.84 (1H) d (1.3)		100.5	5.84 (1H) d (1.2) 5.83 (1H) d (1.2)	
OMe (7)	59.1	3.96 (3H) s ^d^	H_2_-6	59.1	3.97 (3H) s	H_2_-6
OMe (2)	57.7	3.31 (3H) s				
COOCH_3_ (1)	21.6	2.22 (3H) s		21.3 ^d^	2.10 (3H) s ^d^	H-1, H-2
COOCH_3_ (2)				21.3 ^d^	2.10 (3H) s ^d^	H-1, H-2
COOCH_3_ (1)	170.9		H-1, COOCH_3_	170.1		H-1, H-2, COOCH_3_ (1)
COOCH_3_ (2)				170.4		H-2, COOCH_3_ (2)

^a^ The chemical shifts are in δ values (ppm) from TMS; ^b^ 2D ^1^H, ^1^H (COSY) ^13^C, ^1^H (HSQC) NMR experiments delineated the correlations of all the protons and the corresponding carbons; ^c^ Multiplicities were assigned by DEPT spectrum; ^d^ Overlapped signals.

**Table 2:** ^1^H-, ^13^C-NMR and HMBC spectroscopic data of sarniensinol **2** ^a,b^.

No.	^13^C ^c^	^1^H (*J* in Hz)	HMBC
2	55.4 t	3.21 (1H) ddd (9.4, 9.1, 8.5) 2.50 (1H) ddd (9.4, 18.5, 5.0)	H_2_-3, N-Me
3	39.7 t	2.35 (1H) ddd (13.5, 8.5, 5.0) 2.14 (1H) ddd (13.5, 9.1, 18.5)	H_2_-2, N-Me
3a	50.3 s		H_2_-2, H_2_-3, H-4, H-5, H-6
4	137.1 d	5.83 (1H) br s ^d^	H_2_-3
5	124.4 d	5.83 (1H) br s ^d^	H-6, H_2_-7
6	72.2 d	3.89 (1H) dd (5.7, 10.3)	H-5, H_2_-7, OMe
7	26.6 t	2.20 (1H) dd (11.3, 5.7) 1.56 (1H) dd (10.3, 11.3)	H-5, H-6
7a	70.8 d	2.67 (1H) br s	H_2_-2, H_2_-7, N-Me
1′	129.7 s		H_2_-3, H-6′, CH_2_OH
2′	132.5 s		H-3′, CH_2_OH
3′	111.1 d	6.93 (1H) s	CH_2_OH
4′	146.2 s		H-3′, H-6′, OCH_2_O
5′	147.0 s		H-3′, H-6′, OCH_2_O
6′	109.1 d	6.95 (1H) s	H-3′
OCH_2_O	101.2 t	5.93 (2H) br s	
CH_2_OH	63.0 q	4.77 (1H) d (12.0) 4.69 (1H) d (12.0)	H-3′
OMe	55.8 q	3.39 (3H) s	H-6
N-Me	40.4 q	2.39 (3H) s	H_2_-2
OH		3.65, br s	

^a^ The chemical shifts are in δ values (ppm) from TMS; ^b^ 2D ^1^H, ^1^H (COSY) ^13^C, ^1^H (HSQC) NMR experiments delineated the correlations of all the protons and the corresponding carbons; ^c^ Multiplicities were assigned by DEPT spectrum; ^d^ Overlapped signals.

**Table 3:** The initial 24-h mortality testing of compounds **1**–**8** against 1st instar *Ae. aegypti* larvae (*n* = 3).

Compounds	Percent Mortality			
	1 μg/μL	0.5 μg/μL	0.25 μg/μL	0.1 μg/μL
Crinsarnine (**1**)	0	0	0	0
Sarniensinol (**2**)	7 ± 11	0	0	0
Bowdensine (**3**)	13 ± 11	0	0	0
Hippadine (**5**)	7 ± 11	0	0	0
1-*O*-Acetyl-lycorine (**6**)	0	0	0	0
Sarniensine (**4**) ^A^	100 ± 0	80 ± 0	60 ± 0	20 ± 0
3-Epimacronine (**7**) ^A^	60 ± 0	40 ± 0	20 ± 0	0 ± 0
Tazettine (**8**) ^A^	20 ± 0	0 ± 0	0 ± 0	0 ± 0

Positive control permethrin at 6.33 pg/μL resulted in 53% ± 11% mortality and at 47.4 pg/μL resulted in 100% ± 0% mortality; negative control solvent control (DMSO) had 0 mortality. ^A^ Compounds **4**, **7** and **8** were previously reported [15].

**Table 4:** Initial 24-h mortality testing of compounds **1**–**8** against adult female *Ae. aegypti* (*n* = 3).

Compound	Mortality (%) 5 μg/mosquito	LD_50_ ** ± SE (μg/mosquito)	95% CI	R^2^
Crinsarnine (**1**)	97 ± 6	2.29 ± 0.049	(2.41–2.17)	0.9735
Sarniensinol (**2**)	33 ± 6			
Bowdensine (**3**)	33 ± 6			
Hippadine (**5**)	33 ± 6			
1-*O*-Acetyl-lycorine (**6**)	23 ± 6			
Sarniensine (**4**) ^A^	93 ± 6			
3-Epimacronine (**7**) ^A^	67 ± 6			
Tazettine (**8**) ^A^	23 ± 6			
Untreated	0			
Solvent Control (acetone)	0			
0.15 ng Permethrin	37 ± 6			
0.23 ng Permethrin	63 ± 5			
2.37 ng Permethrin	100 ± 0			

^A^ Compounds **4**, **7** and **8** were previously reported [15]; ** After the primary screening of the compounds, compounds showing mortality >80% were further subjected to LD_50_ dose-response bioassays.
